# Aerogels for Biomedical, Energy and Sensing Applications

**DOI:** 10.3390/gels7040264

**Published:** 2021-12-14

**Authors:** Muhammad Tayyab Noman, Nesrine Amor, Azam Ali, Stanislav Petrik, Radek Coufal, Kinga Adach, Mateusz Fijalkowski

**Affiliations:** 1 Department of Machinery Construction, Institute for Nanomaterials, Advanced Technologies and Innovation (CXI), Technical University of Liberec, 461 17 Liberec, Czech Republic; nesrine.amor@tul.cz; 2 Department of Materials Engineering, Faculty of Textile Engineering, Technical University of Liberec, 461 17 Liberec, Czech Republic; azam.ali@tul.cz; 3 Department of Advanced Materials, Institute for Nanomaterials, Advanced Technologies and Innovation (CXI), Technical University of Liberec, 461 17 Liberec, Czech Republic; stanislav.petrik@tul.cz (S.P.); Kinga.Adach@tul.cz (K.A.); mateusz.fijalkowski@tul.cz (M.F.); 4 Department of Science and Research, Faculty of Health Studies, Technical University of Liberec, 461 17 Liberec, Czech Republic; radek.coufal@tul.cz

**Keywords:** aerogels, silica aerogels, porous materials, catalysts, sensors

## Abstract

The term aerogel is used for unique solid-state structures composed of three-dimensional (3D) interconnected networks filled with a huge amount of air. These air-filled pores enhance the physicochemical properties and the structural characteristics in macroscale as well as integrate typical characteristics of aerogels, e.g., low density, high porosity and some specific properties of their constituents. These characteristics equip aerogels for highly sensitive and highly selective sensing and energy materials, e.g., biosensors, gas sensors, pressure and strain sensors, supercapacitors, catalysts and ion batteries, etc. In recent years, considerable research efforts are devoted towards the applications of aerogels and promising results have been achieved and reported. In this thematic issue, ground-breaking and recent advances in the field of biomedical, energy and sensing are presented and discussed in detail. In addition, some other perspectives and recent challenges for the synthesis of high performance and low-cost aerogels and their applications are also summarized.

## 1. Introduction

The term aerogel is used for ultralow density and lightweight materials derived from organic, inorganic or hybrid molecular precursors. Aerogels contain 99.9% air in their structure, which makes a chain of porous network. Therefore, aerogels are solids similar to gel that contain air pockets [1]. Scientifically, aerogels are highly porous, light-weight and unique solid-state structures composed of three dimensional (3D) interconnected networks filled with a huge number of air pores [2]. These air-filled pores enhance the physicochemical properties and the structural characteristics in macroscale as well as integrate typical characteristics of aerogels, e.g., low density, high porosity and some specific properties of their constituents [3,4]. These extraordinary and attractive characteristics endow aerogels as a first choice in highly sensitive sensing and energy applications, e.g., biosensors [5,6], gas sensors [7], pressure strain sensors [8], supercapacitors [9], catalysts [10,11], energy storage [12,13], piezoelectric [14], thermal insulators [15,16] and ion batteries [17]. The term aerogel was coined by Kistler in the early 1931 to describe his synthesized gels, where the liquid was replaced with a gas without collapsing the solid gel structure [18]. In the beginning, there was limited research work on aerogels after their first discovery; the term aerogel had a rebirth in the 1970s for the popularity of sol-gel synthesis methods and the application of aerogels for storing rocket fuels. Afterwards, significant efforts were made to simplify the synthesis methods, especially drying, for low-cost and facile synthesis of aerogels. This opened the gateway for a variety of aerogels, due to their open structure and light-weight characteristics in different fields of applications.

**Figure 1:**
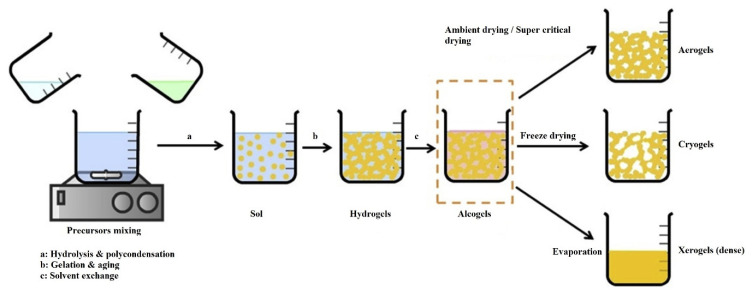
A schematic illustration of the conventional synthesis method of aerogels. Reprinted with permission from [24].

The global development and continuous improvement in daily lifestyle have posed a burden on natural energy sources such as fossil fuels. The scarcity of these traditional resources and their impact on the surrounding environment has created challenges for mankind. In order to decrease this burden, research work has been carried out in different directions. One such direction is to rely on renewable energy sources such as solar energy. The conversion of concentrated solar energy into thermal or electricity is under the consideration of the scientific community. In this regard, porous materials provide a good solution for improving the heat transfer characteristics in solar systems [19,20,21]. Porous materials with their exceptional characteristics, i.e., low cost, light-weight and significant potential to augment thermal properties, have received considerable attention. These materials are used in different industrial applications, e.g., thermal insulation devices, adsorption devices, energy storage devices, geothermal devices and evaporating devices, due to their porosity and solid matrix structure [22]. The formation of a 3D network is a key factor in the synthesis of aerogels, with better durability, stability and higher porosity. Wet chemical methods are known as conventional methods to fabricate different types of aerogels, including inorganic, organic and hybrid, and sol-gel synthesis is one of the most prominent wet chemical methods for aerogel preparation [23]. Sol-gel method is generally completed by mixing precursors, followed by hydrolysis, polycondensation and then gelation, aging and finally drying by appropriate methods (ambient drying, super critical drying, freeze-drying or lyophilization). In sol-gel synthesis, each step is realized by the relevant variables, i.e., pH of the solution, concentration of precursors, time, temperature and type of solvents in order to fine-tune the final properties of the aerogels. Acidic and basic catalysts are used during hydrolysis and polycondensation reactions, depending on the materials to be catalyzed; the reaction is catalyzed. The drying of the gel is the key step among all the mentioned steps, which determines the overall efficiency and final properties of the aerogels. To date, the most intensive and significantly used drying methods for the preparation of aerogels are ambient drying (evaporation), freeze-drying (lyophilization) and super critical drying. Figure 1 shows a schematic illustration of the conventional aerogel synthesis procedure [24].

The procedure illustrated above has been mainly used and reported for aerogels. All other aerogel production techniques are either derived or altered from this procedure. Many researchers synthesized various types of aerogels, including organic aerogels [25], silica-based aerogels [26], metallic aerogels [27], carbon aerogels [28], chalcogenide aerogels [29] and hybrid aerogels [30] by this method [31,32]. Intensive research studies were carried out on the applications of aerogels in the past few years, indicating the great field of application for aerogels. There are many review papers available on the applications of aerogels; however, this work is related to aerogels made of different materials, i.e., carbon, gold, silver, zinc, titanium, cobalt and cellulose for biomedical, energy and sensing applications. We compared the significance of other materials, especially cellulose, carbon and some metals, with silica aerogels and demonstrated their importance for biomedical, energy and sensing applications. Among other potential applications, sensors have gained tremendous attention because they are vital in health monitoring, public safety, environment and industries, and aerogels have demonstrated their worth as an excellent material for sensors. The aim of this paper is to provide an up-to-date study on the applications of aerogels in energy, environment and sensing forms.

## 2. Properties and Classification of Aerogels

Aerogels are considered ultra-high nano-porous structures. These structures enable aerogels to be optimal candidates for many sensitive fields of applications, including energy, sensors and thermal protection. However, aerogels display mostly brittle behavior; for thermal insulation of complex geometrical structures, this type of mechanical behavior is not suitable because flexibility of the material is a primary concern. To allow certain flexibility in the aerogel structures, research efforts had been made and materials synthesized, which can be divided into two main categories, i.e., induced flexible and inherently flexible aerogels. Most of the flexible aerogels that are commercially available belong to induced-flexible aerogels, where the flexibility of the structure is induced by additives and components other than the aerogel material itself. Aerogels are classified into organic, inorganic and hybrid aerogels according to the type of precursor used during the synthesis of aerogels. However, according to their surface chemical properties, they are categorized into hydrophobic and hydrophilic aerogels. Table 1 shows the structural characteristics of aerogels reported by various studies. As shown in the table, particle size and surface area of the aerogels range between 2 nm to 5 mm and 300 to 1100 m^2^·g^−1^, respectively. The density and porosity range between 40–350 kg·m^−3^ and 85–99.9%, respectively. The pore diameter varies from 1–109.5 nm and thermal conductivity between 0.01–0.02 W·m^−1^·K^−1^, respectively.

It is revealed from Table 1 that most of the structural characteristics primarily depend on synthesis route and type of precursor used. Silica aerogels are synthesized at the earliest and classified as inorganic aerogels. Silica aerogels are widely studied due to their facile synthesis and commercial development and are considered as a standard form of aerogels for comparison with newly developed aerogels. The synthesis process of silica aerogels consists of the following steps: silica gel is prepared as a first step and then the gel is processed for aging and drying. Ambient drying and supercritical drying processes are mostly followed during the synthesis of aerogels. However, the lateral method is challenging due to hazards and high cost in perceiving the large-scale production of aerogels. In a previous study, Maleki et al. reviewed the synthesis of silica aerogels with different methods and stated that ambient drying is a facile and cost-effective method to synthesize the composites of aerogels. In addition, post gelation and high pressure makes ambient drying more suitable for the bulk production of aerogels [46].

Another important type of aerogels is metal-based aerogels that are indispensable for their excellent electrical conduction properties. Metallic aerogels have gained intense attention in recent years due to their higher surface area and ultralow densities. Researchers worked with various type of metals, i.e., gold, silver, copper, titanium, zinc, nickel, cobalt and successfully fabricated metal aerogels with tunable surfaces, structural and electrical properties [47,48,49,50]. In an experimental study, Qian et al. reported the synthesis of ultralight gold aerogel monoliths with tunable pore densities and structures. Different solvents and their suspension are crucial parameters for the systematic tuning of monolithic gold aerogels with enhanced densities and pore architectures [51]. In a previous study, Qian et al. synthesized ultralight silver aerogel monoliths with excellent conduction properties and tunable densities via silver nanowires. Silver nanowires were used as building blocks, and the freeze-casting method followed by sintering was used as the fabrication route for silver aerogel monoliths [52]. Yan et al. reported a three-dimensional printing technique and freeze casting method for the fabrication of metallic aerogels. The results demonstrated that by using these techniques, the densities are controllable and high electrical conductivity is achieved [53]. In a different study, Xu et al. fabricated ultralight flexible pressure sensors with the help of copper nanowires. Copper nanowires were assembled into copper aerogels via the one-pot method. The fabricated pressure sensors demonstrated excellent results for sensitivity, tunable pore architecture and ultralow density [54].

The cost and properties of silica aerogels mainly depend on the fabrication methods. The precursors mainly used for the synthesis of silica aerogels are sodium silicate and various types of silanes, e.g., tetramethoxysilane, tetraethoxysilane and polyethoxydisiloxane. Due to their hazardous effects and higher cost, silanes are not commercially used for the fabrication of silica aerogels. However, for bulk production and cost effectiveness, sodium silicate is utilized as a cheaper precursor for the synthesis of silica aerogels [55]. In a previous study, Carlson et al. reported that the synthesis of silica aerogels with sodium silicate precursor is 7.7–13.5 times more cheaper than silanes precursors [56]. We came to know that silica aerogels are brittle in nature and additives (polyethylene glycol) play an essential role for maintaining the pore volume size and mechanical properties. The addition of polyethylene glycol in the synthesis of aerogels control the strength of solution matrix, which means a lower concentration of polyethylene glycol strengthens the solution matrix and vice versa. In addition, the concentration of hydrophilic and water-soluble polymers adjusts the pore size of silica aerogels.

Due to their remarkable properties and structure, graphene oxide (GO) has been investigated and used in the synthesis of aerogels. GO improves the porosity during the interaction with silica matrix, restricts the transfer of heat and enhances the thermal properties 1.5 times more than pure silica aerogels. In addition, GO enhances the mechanical properties besides the augmentation in the thermal stability of the composite [57]. Lei et al. worked with silica aerogels and added GO as a nanofiller during the synthesis of GO/SiO_2_ composites aerogels. They reported that GO improves the thermal insulation and mechanical properties of the aerogels. The positive influence of GO was due to the homogenous distribution of GO inside the silica matrix and the interfacial interaction between silica and GO nanosheets [58].

In a similar manner, the inclusion of fibrous material during the synthesis of aerogels enhances the thermal and mechanical properties. Patil et al. investigated the effect of carbon nanotubes, glass fibers and graphene nanosheets during the synthesis of silica aerogels and compared the results with virgin silica aerogels. The results revealed that aerogels composed of carbon nanotubes, glass fibers and graphene nanosheets demonstrate significant augmentation in tensile strength by nine, three and eight times and 11.5, 3.5 and 9.5 times augmentation in elastic modulus than standard silica aerogels, respectively [59]. Li et al. conducted many studies on silica aerogels composed of aramid fibers to enhance thermal and mechanical properties. The results revealed that silica aerogels with aramid fibers possess lower bending modulus with exceptional flexibility. The compressive strength increased up to 1.2 MPa with extremely low thermal conductivity 0.0227 ± 0.0007 W·m^−1^·K^−1^ [60,61].

We came to know a fact through literature review that silica aerogels are mostly used as commercial thermal insulating materials due to their economic raw materials and facile drying process. The ambient drying process is mostly used for the commercial production of silica aerogels due to its economic benefits. In general, aerogels are extremely high thermally insulating materials with poor mechanical properties that may be improved or adjusted by the inclusion of different additives, i.e., fibrous materials, carbon nanotubes and graphene, during the synthesis mechanism.

## 3. Applications of Aerogels

Aerogels are special materials that have an enormous diversity of outstanding physicochemical properties, including mechanical, physical and chemical properties, and therefore many applications have developed with aerogel utilization. Some of the most sensitive applications are catalysis, thermal insulation, electrodes, solar thermal energy systems, waste engine oil, oil spill cleaning, drug and protein delivery, medical implantable devices and supercapacitors. This section presents the recent advanced technical applications of aerogels in different fields, including biomedical engineering, energy, environment and sensors.

### 3.1. Aerogels for Biomedical Engineering

Aerogel materials has proven potential applications in biomedicines and attracted great attention in recent years. Aerogels have been applied in different applications, such as tissue engineering, drug and protein delivery, implantable medical devices, bone grafting, biosensing and blood sorption [62,63,64]. This section introduces various recent applications of aerogels in biomedical engineering.

**Figure 2:**
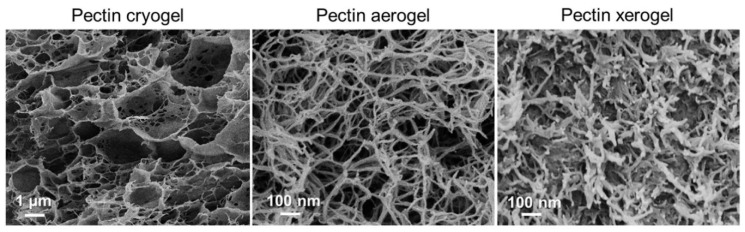
SEM micrographs of pectin cryogels, aerogels and xerogels. Reprinted with permission from [68].

**Figure 3:**
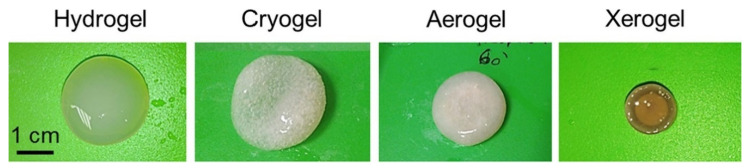
Practical samples of a pectin hydrogel, cryogel, aerogel and xerogel. Reprinted with permission from [68].

Aerogel has been widely used in tissue engineering for the regeneration of different types of tissues, such as bones, skin, blood vessels, and cartilages. Muñoz-Ruíz et al. proposed and evaluated the collagen-alginates aerogel for the regeneration of different tissues using bio-based materials, in order to solve the problem of the potential complications related to autografts. The results revealed that the induced properties after drying are responsible for these changes. The aerogel microstructure was very stable and composed of highly porous 3D interconnected networks that helps in cell attachment [65]. Osorio et al. modified cellulose nanocrystal aerogels and used them as viable bone tissue scaffolds. The experimental results demonstrated that cellulose-based aerogels are porous and flexible and facilitate bone growth after their implant in bone defects. In addition, the aerogels demonstrated an increase in cell metabolism [66]. Reyes-Peces et al. presented a hybrid aerogel structure composed of chitosan and silica for bone tissue engineering. They synthesized the hybrid aerogels by the sol-gel method followed by supercritical CO_2_ drying. They used glycidoxypropyl trimethoxysilane (GPTMS) as the crosslinking or coupling agent, and tetraethylortosilicate (TEOS) as silica precursor. The results of in vitro study demonstrated that the proposed hybrid chitosan silica aerogel composite structure provides a durable substitute in bone tissue engineering for cells recruitment and maturation by inducing an excellent osteoblast response [67]. Groult et al. investigated the influence of the drying process on the structural and release properties of pectin hydrogels, aerogels, cryogels and xerogels. They prepared the porous pectin aerogels, xerogels and cryogels using super critical drying, freeze-drying and evaporative drying under low vacuum. They investigated and compared the kinetics of drug release from all the mentioned materials, including hydrogel. Scanning electron microscopy (SEM) images illustrate that the drying method has a noteworthy influence on different properties of the pectin networks, as shown in Figure 2. Shrinkage was observed for pectin cryogels that might originate upon immersion of the sample into nitrogen for the freezing of water prior to sublimation. Due to this, samples made with the freeze-drying method display very low density. Figure 3 shows the experimental samples with the dry core in pectin aerogels, which confirm the slow solvent transport through the dry system, in contrast with the pectin hydrogels and cryogels [68].

Rostamitabar et al. produced cellulose aerogel fibers under the super critical CO_2_ drying method and tested their samples on the drug model. The surface morphology and structural properties of the synthesized aerogels were characterized by different spectroscopies. In addition, thermal stability, mechanical properties and drug release assessments were also performed. The results revealed that fibrous structures were able to absorb excessive amount of moisture and due to their open porous structure, aerogels released the drug immediately and demonstrated non-toxic behavior [69]. In another study, Marco et al. produced polysaccharide-based aerogels, which were further used as carriers for drug delivery systems. They analyzed their samples on the basis of the life cycle assessment and from an environmental point of view to minimize total emissions. Aerogels of starch were synthesized under the following three steps: (1) Prepare the gel using an aqueous solution; (2) replace the water by alcohol to develop alcogels; (3) use of the super critical CO_2_ drying process to obtain bio aerogels. The experimental results demonstrated that the carcinogens and mineral extraction were mainly the affected categories as a consequence of the high energy consumption in the drying step; in addition, the respiratory organics were infected due to the ethanol used in the alcogel formation [70].

**Figure 4:**
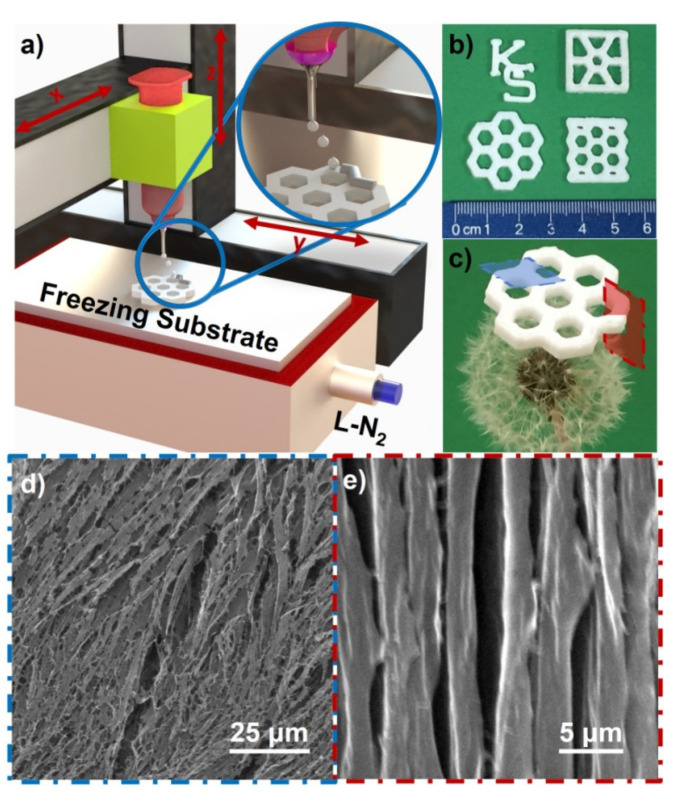
(**a**) A schematic illustration of 3D freeze-printing process. (**b**) Different geometries of 3D cellulose aerogels. (**c**) Cellulose aerogel with honeycomb shape standing on a dandelion. (**d**) SEM micrograph displaying the top surface of 3D freeze-printed aerogel. (**e**) SEM micrograph displaying the cross-sectional surface of 3D freeze-printed aerogel. Reprinted with permission from [72].

Recently, Saadatnia et al. developed conductive aerogel films for the monitoring of electrophysiological properties and proposed a novel model of wet electrode. The electrodes were composed of cellulose nanocrystals and multi-walled carbon nanotube. Cellulose nanocrystals were used as biopolymers and carbon nanotubes as conductive fillers. The produced electrode is featured to investigate the electrical, chemical, water absorption and mechanical properties. The fabricated model has very high performance, which makes it effective for wet electrode applications, thanks to the high percentage of water absorption due to its hydrophilicity and porosity. In addition, the proposed model guarantees the multiple use of a fabricated electrode for different applications, such as remote and long-term monitoring of patients, and for different electrophysiological monitoring devices, e.g., electroencephalography (EEG) and electrocardiography (ECG) [71]. In another study, Tetik et al. synthesized cellulose-based aerogels with excellent 3D geometries and shapes with overhang properties. They determined the impact of the used method for the improvement of the mechanical properties of synthesized aerogels. In addition, they evaluated the bonding strength to augment the stability of aerogels in order to make them feasible for tissue engineering and biomedical applications. Figure 4 shows the essential steps involved in the making of aerogels used in materials and methods as well as the results of SEM observations [72].

### 3.2. Aerogels for Energy

Aerogels have the advantages of biodegradability and low density that make them a good choice for solving serious environmental problems. Researchers have proposed and developed many techniques that produce green and sustainable electrodes, based on aerogels, as a solution for pollution and other environmental concerns [73,74,75]. In an experimental study, Strobach et al. reported the synthesis of optically transparent and thermally insulating monolithic silica aerogels with high solar transparency, especially developed for the solar thermal receiver. They elaborated the effects of annealing at different annealing temperatures. They explained that the structures and properties of aerogels could be controlled by controlling the temperature. In addition, the time and temperature of annealing were helpful in the optimization of aerogels for better optical and thermal properties. The results elucidated good prediction performance of aerogels in solar thermal applications [76].

**Figure 5:**
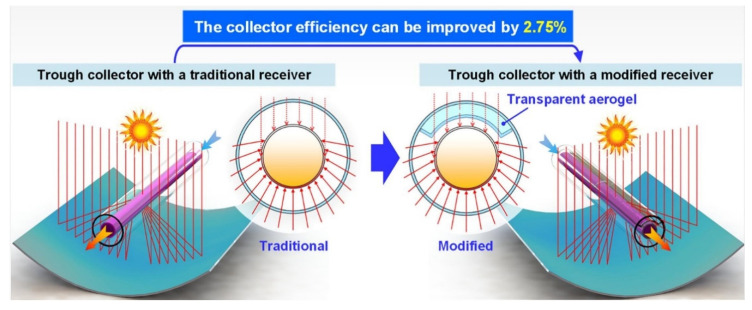
Traditional evacuated receiver and evacuated receiver modified by aerogels. Reprinted with permission from [77].

Li et al. introduced a modified evacuated receiver with solar transparent aerogels in order to improve the receiver performance. In addition, they developed and validated an optical-thermal model for modeling the energy transfer in the collector. Figure 5 exhibits the traditional evacuated receiver that was chosen as a standard model and the modified receiver that was designed with the addition of aerogels at an angle of 120° at a directly illuminated region. The results revealed that the effects of aerogels on the modified receiver are significant. Aerogels coating’s emittance greatly affects the receiver performance. The optimal efficiency was achieved by adding aerogels with opaque insulation [77].

Recently, Han et al. prepared thermally insulated aerogels using TiO_2_ and chitosan under combined thermal reduction and freeze-casting methods. Experimental results demonstrated that the produced aerogels have excellent mechanical properties, thermal insulation behavior and good high-temperature service performance [78]. Lui et al. synthesized novel and homogeneous TiO_2_/SiO_2_ aerogel composites with synchronous sol-gel method. The proposed technique provides hydrophobic properties achieved by pre-heat treatment and surface modification. The thermal conductivity test, structure characterization, energy saving simulation calculation and infrared radiation heat transfer calculation of TiO_2_/SiO_2_ aerogel composite were executed. Therefore, the thermally insulated properties for steam pipe application that works for high temperatures were obtained accordingly. The obtained results of TiO_2_/SiO_2_ aerogel composite were compared with hybrid glass fiber/SiO_2_ composites. The results demonstrated that glass fiber/TiO_2_/SiO_2_ aerogel composite had better results for thermal conductivity than others. The energy-saving effect of the glass fiber/TiO_2_/SiO_2_ aerogel composite demonstrated excellent performance in saving oil consumption silica aerogels [79].

**Figure 6:**
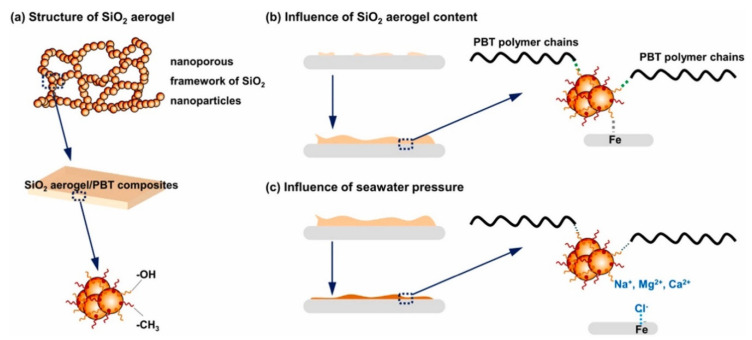
Application of silica aerogels in sea water, (**a**) structural evolution of silica aerogels, (**b**) the influence of silica content, (**c**) seawater pressure on silica aerogels and poly butylene terephthalate composites. Reprinted with permission from [91].

The role of aerogels for energy applications, especially supercapacitors and batteries, has been increasing day by day. Long et al. introduced a novel method for the fabrication of N_2_ doped carbon aerogels for supercapacitors. They prepared the biomass-derived carbon aerogel using glucose, cellulose nanofibers and dicyandiamide as precursors. The multifunctional materials were validated on flexible electronics, sensors and energy storage/conversion devices, i.e., supercapacitors, where they demonstrated excellent thermal and mechanical properties [80]. In another study, Lui et al. reported the synthesis of tungsten oxide (WO_3_) nanoparticles and their homogenous dispersion and deposition on carbon-based aerogels for the development of supercapacitors. Experimental results demonstrated that the addition of WO_3_ nanoparticles enhances the capacitance with a reduction in size and provides an exponential increase for capacitance values; however, during cycling, carbon aerogels facilitate charge transfer. The proposed method achieved a high value of capacitance in comparison with other kinds of nanomaterials deposited on carbon aerogels for supercapacitor applications [81]. Carbon-based aerogels are generally famous for lightweight and long-lasting batteries with excellent charge/discharge capabilities. Muniyandi et al. worked with carbon-based hybrid aerogels for high performance lithium-ion batteries. They successfully synthesized Li_2_FeSiO_4_/C aerogels with sol-gel method and supercritical drying. The electrode kinetics and storage performance of the synthesized aerogels were characterized by electrochemical impedance spectroscopy, cyclic voltammetry and galvanostatic charge discharge methods. The results demonstrated that the fabricated electrode delivers 140 mA·h·g^−1^ discharge capacity for 130 cycles [82]. In another experimental study, Chen et al. incorporated Fe_3_O_4_ nanoparticles during the synthesis of carbon nanotubes aerogels that significantly enhanced the electron transport, ion diffusion and reduced volume expansion of lithium-ion batteries. Moreover, the results demonstrated that the fabricated anode delivers extraordinary charge/discharge (reversible) capacity after 100 cycles [83]. Jiang et al. worked with boron nitride nanomaterial-based aerogel composites to achieve low thermal conductivity. They reported the fabrication of aerogel-based thermoelectric batteries with excellent durability, no maintenance, long life and high reliability [84]. Some other applications of aerogels are in the form of catalysts, construction and building materials that possess the potential for environmental remediations [85,86,87,88,89]. Golder et al. reported the significance of aerogels in the development of insulating materials for construction and buildings. They deposited translucent aerogel glazing systems in the walls and windows of different buildings for better insulation performance. The results demonstrated that the deposition of aerogels in the wall and window insulation and glazing systems has significant potential to reduce energy consumption and cost of buildings [90]. Qi et al. reported the tribology behavior of silica aerogels reinforced with polybutylene terephthalate for a deep-sea environment. They evaluated the tribological properties, thermal and mechanical properties, absorption of binary nanocomposites and seawater wettability. Their results demonstrated that the incorporation of silica aerogels increase the wear-resistance, seawater repellency and thermal stability. However, they decrease the acceleration of seawater absorption, mechanical strengths and wear rate of polybutylene terephthalate. A schematic diagram of silica aerogels for sea environment is illustrated in Figure 6 [91].

### 3.3. Aerogels for Sensors

Aerogels and aerogel-based composite materials have strong mechanical strength, excellent flexibility, high porosity, lightweight and excellent durability. These characteristics attract the researchers interests and fulfill the requirements for the fabrication of various types of sensors, e.g., gas sensors, electrochemical sensors, pressure sensors, humidity sensors, flexible sensors and tactile sensors. With the advancement in science and research, gas sensors have achieved significant importance in many fields for the detection of explosive and toxic gases as well as the gases for disease diagnosis [92,93]. Resistive types of gas sensors are mostly used due to their facile fabrication, low cost and easy operation. In resistive sensors, the sensing operation takes place on the surface of the active sensing material and the conductivity changes during the absorption of the gas molecules on the surface of the active sensing layer. Aerogel-based sensors have two major advantages, i.e., first, a high specific surface area and surface-to-volume ratio that provides sufficient surfaces for the adsorption of gas molecules; second, a 3D porous interconnected structural network that provides a stable and fast transport channel for the diffusion of gas molecules. Thus, aerogel-based sensors demonstrate fast recovery rate, low detection limit, high sensitivity and fast response rate. Table 2 shows some recent work on aerogel-based gas sensors.

Alizadeh et al. reported the fabrication of an ammonia gas sensor with the help of graphene hydrogels characterized with ppb level determination capability. They followed the hydrothermal method for the synthesis of 3D graphene aerogels. The mechanism of sensing was related to the variation in electrical resistance. The fabricated device was highly efficient and capable of sensing ammonia gas in a reversible manner at ambient temperature under a short span of time [101]. Gao et al. fabricated gas sensors based on zinc oxide and reduced the graphene oxide aerogel composites for the detection of nitrogen dioxide (NO_2_). The obtained results reveal that after the freeze-drying method, aerogels have a regular cylindrical shape with larger pore sizes; however, zinc oxide was homogenously distributed on the surface of graphene oxide and provides a solid network of crosslinking. In addition, the results demonstrated that the fabricated sensor provides a quick response for NO_2_ detection with a fast recovery rate and good reproducibility [102]. Bibi et al. reported the fabrication of carbon aerogels and polyaniline-based gas sensor for the detection of hydrogen sulphide (H_2_S) gas. They explained that aerogel-based composites were first applied on the interdigitated electrode made of glass. Carbon aerogels were dispersed with the spin-coating method on the electrode for gas sensing [103].

**Figure 7:**
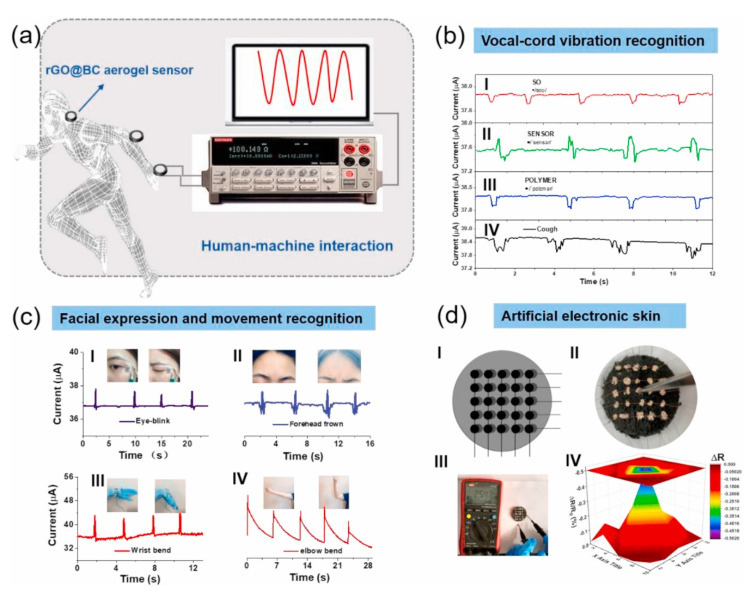
(**a**) Illustration of reduced graphene oxide-based aerogel-sensing platform, (**b**) Vocal-cord vibration recognition, (**c**) facial expression and body movement monitoring, and (**d**) aerogel-based artificial skin. Reprinted with permission from [106].

The pressure and strain sensors convert the applied pressure change and deformation into the electricity of an object. These sensors have demonstrated their potential in different fields, especially in wearable health monitoring devices and artificial skins. The sensing mechanisms of any pressure and strain sensor are based on piezo resistivity, capacitance and piezoelectricity. Piezoresistive pressure and strain sensors are the most promising sensors, among other types of pressure sensors, and they are widely studied due to their facile read-out system and easy structures. In addition, the aerogel-based piezoresistive pressure and strain sensors have enough tolerance for large deformation and high elasticity; therefore, they provide a large sensing range. In an experimental study, Zhu et al. prepared a sensitive piezoresistive sensor, based on reduced graphene oxide and carbon nanotube aerogels, by using a hydrothermal redox method for human motion detection. Furthermore, they proposed that the fabricated sensor provided good stability, fast response time, high sensitivity and a wide working range [104]. Cao et al. reported the synthesis of polyacrylonitrile nanofiber-reinforced graphene aerogels for piezoresistive-sensing applications. In this method, nanofibers worked as scaffolds for the graphene network and provide a 3D interconnected hierarchical microstructure. The results demonstrated excellent compression resilience, fast response time, perfect sensing durability and structural stability. Furthermore, the fabricated sensors were able to evaluate real time movements of the wrist, fingers, wrist pulse and knee joints at a good sensitivity [105]. Wei et al. reported the fabrication of the pressure sensor based on graphene/biomass hybrid aerogels. A facile and green strategy was used to fabricate these types of sensors by the effective reduction of graphene oxide through bacteria cellulose and caffeic acid. The fabricated sensor demonstrate fast response, high sensitivity and excellent reproducibility. Sensors exhibit an integrated performance of ultralow limit of detection, high sensitivity and fast responses, and clearly detect the subtle strain and monitor physical human motions. Figure 7 shows the application of fabricated sensors during the capturing of human motion [106].

**Figure 8:**
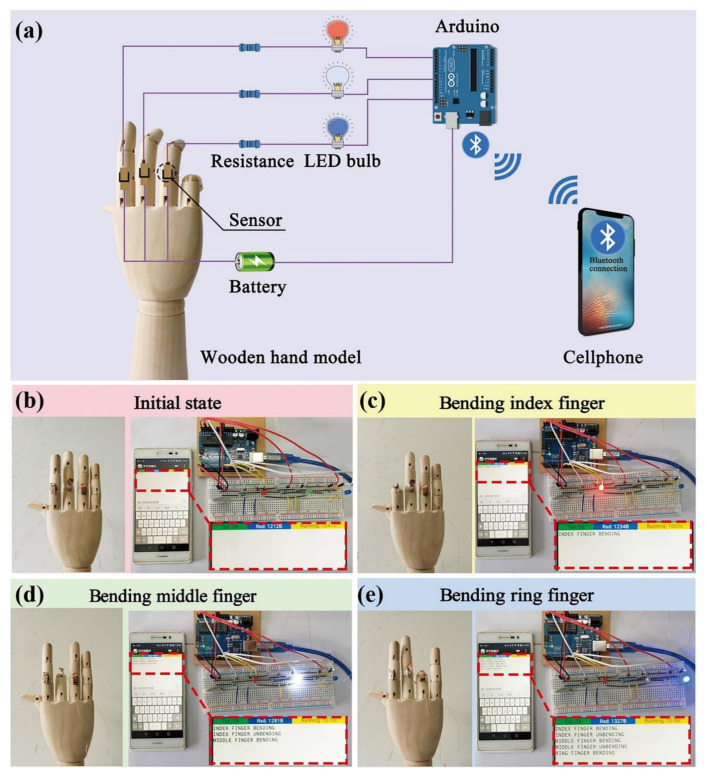
(**a**) Illustration of Arduino microcontroller connected to a circuit for finger movement detection, sending messages to a cellphone through Bluetooth connection. Photographs of the wooden hand model (**b**) in the initial state, (**c**) bending index finger, (**d**) bending middle finger, (**e**) bending ring finger and the corresponding photographs of the cellphone displaying messages from Arduino microcontroller and LED bulbs emitting red, white and blue lights, respectively. Reprinted with permission from [108].

Carbon-based aerogels are widely studied for their electrical properties, good compressibility and high porosity as piezoresistive sensors. Bi et al. reported the fabrication of electrodes made of carbon aerogels clustered on a carbon ball. The main purpose of the proposed technique is to develop a electrochemical sensor from the biomass of taros. The results demonstrated a high electrochemical activity performance and the proposed method revealed its potential as a powerful electrode for the fabrication of multi-functional electrochemical sensors for practical applications [107]. Yang et al. fabricated superhydrophobic and conductive aerogels with honeycomb-like microstructures under directional freeze-drying methods for the piezoresistive pressure sensor. The fabricated sensor provides a wide detection range, excellent electrical repeatability, stability and fast respond times. The fabricated sensor was used to evaluate the motion of the human body, where it demonstrated stable work under humid or sweaty environments. In addition, the fabricated sensor detected real-time movements of finger joints, as illustrated in Figure 8 [108].

## 4. Summary and Future Direction

❖ In this study, a brief history and the applications of aerogels based on their classification and synthesis methods are reviewed and discussed. Aerogels are considered as excellent candidates for biomedical, energy, environment and sensing applications, and especially for high-performance sensors, where high sensitivity is required. Due to their structural characteristics and other properties, including having a highly porous structure, high specific surface area and other specific features provided by the aerogel network, aerogels are suitable for many applications. Therefore, based on the discussion, it is concluded that the combination of low dimensional active building blocks induces fascinating properties in the resulting aerogels, which display satisfactory performances in multiple applications. However, there are many challenges that still need to be addressed.
❖ The economic and bulk production of high-quality aerogels is still a major issue that needs to be solved. Efforts have been made to simplify the synthesis mechanism in order to scale up and to reduce cost. Therefore, freeze drying (lyophilization), modified super critical drying and ambient drying methods were used. However, during the drying conditions, it was not easy to completely maintain the microstructure of the gel, therefore, damage frequently occurred.
❖ Surface modification techniques employed for ambient drying inevitably causes a negative effect on the performance of aerogels. The overall properties of aerogels become disturbed during surface modification. The discussed studies demonstrate the superiority of aerogels in comparison with powdered materials in different fields. Therefore, aerogels with no structural variation own superior properties and a large-scale production of low-cost aerogels with superior qualities is vital and should be realized.
❖ Maintain the porosity of the structure, especially the microporosity of the aerogel structure under stress during the fabrication of aerogel products, i.e., biosensors, gas sensors, ion batteries and catalysts. During the fabrication of sensors, aerogels are dispersed in solvents in order to get a uniform dispersion and coated on other substrates to make aerogel-based sensors. During this dispersion process, the microporous structures of aerogels are deteriorated and a decrease in mass and energy transfer is observed in the resultant sensor. However, in a comparison, these sensors still behave better and display a much-increased sensing performance than powder-based sensors. It is still believed that if the intrinsic porosity of the aerogel structures is retained well during the fabrication of sensors, the performance of the aerogel-based sensor can further be improved.
❖ The utilization of thin aerogel films covers the microporous structural damage caused during the synthesis of sensors. Aerogels with excellent mechanical features are the prerequisites for the synthesis of aerogel films. Therefore, the mechanical properties of various types of aerogels, e.g., organic, inorganic and hybrid, should be improved. In this matter, the inclusion of additives (supporting materials) is considered a facile way to increase the overall mechanical properties. Although, when the mechanical properties are enhanced and the structure of aerogel is controlled, the extension of this technique remains a challenge for all kinds of aerogels. Therefore, it is compulsory to develop a common method that works for all types of aerogels to enhance their mechanical properties.

## Figures and Tables

**Table 1:** Structural characteristics of different aerogels.

Aerogel Type	Precursor	Surface Area [m^2^·g^−1^]	Density [kg·m^−3^]	Porosity	Pore Size	Thermal Conductivity [W·m^−1^·K^−1^]	Reference
Silica aerogels	C_4_H_12_O_3_Si	600–1000	350	85–99.9%	1–100 nm	0.010–0.020	[33]
Silica aerogels	Na_2_SiO_3_	600–1000	300–350	-	20 nm	-	[34]
Silica aerogels	C_4_H_12_O_3_Si	576	100	>90%	20–100 nm	0.020	[35]
Silica aerogels	Na_2_SiO_3_	366	40–150	>90%	20–100 nm	-	[36]
Silica aerogels	Na_2_SiO_3_	300–400	50–80	98%	20–40 nm	0.016–0.020	[37]
Carbon aerogels	C_3_H_8_N_2_O	300	0.24	99%	-	-	[38]
Carbon aerogels	C_2_H_3_Cl	1600	-	98%	2 nm	-	[39]
Silver aerogels	AgNO_3_	400	27	98%	10–100 nm	-	[40]
Zinc aerogels	ZnC_4_H_6_O_4_	350	-	99%	-	-	[41]
Titanium aerogels	C_16_H_36_O_4_Ti	300	-	98%	20	-	[42]
Cellulose aerogels	Wood	-	-	98%	-	-	[43]
Chitosan aerogels	Chitosan powder	400	-	99%	-	-	[44]
Biomass aerogels	Konjac glucomannan	-	47	95%	-	-	[45]

**Table 2:** Aerogel-based sensors and their performance.

Aerogel Type	Analyte	Response/Recovery Rate	Sensing Range	Detection Limit	References
Graphene aerogel	Ammonia	100 s/500 s	0.02–85 ppm	10 ppb	[94]
Carbon aerogel	Toluene and n-hexane	25 s/20 s	-	-	[95]
Graphene aerogel	NO_2_	116 s/169 s	0.1–1 ppm	50 ppb	[96]
TiO_2_/SiO_2_ aerogels	H_2_S	53 s/74 s	0.5–50 ppm	0.5 ppm	[97]
ZnO/graphene aerogels	NO_2_	132 s/164 s	10–200 ppm	10 ppm	[98]
Silica aerogels/ Carbon quantum dots	NO_2_	-	2–10 ppm	250 ppb	[99]
Silica aerogel film	Humidity	38 s/21 s	20–90% RH	-	[100]
